# APX2 Is an Ascorbate Peroxidase–Related Protein that Regulates the Levels of Plastocyanin in *Chlamydomonas*

**DOI:** 10.1093/pcp/pcae019

**Published:** 2024-03-02

**Authors:** Anna Caccamo, Félix Vega de Luna, Agnieszka E Misztak, Sébastien Pyr dit Ruys, Didier Vertommen, Pierre Cardol, Joris Messens, Claire Remacle

**Affiliations:** Genetics and Physiology of Microalgae, InBios/Phytosystems Research Unit, University of Liège, Chemin de la vallée 4, Liège 4000, Belgium; VIB-VUB Center for Structural Biology, Pleinlaan 2, Brussels 1050, Belgium; Brussels Center for Redox Biology, Pleinlaan 2, Brussels 1050, Belgium; Structural Biology Brussels, Vrije Universiteit Brussel, Pleinlaan 2, Brussels 1050, Belgium; Genetics and Physiology of Microalgae, InBios/Phytosystems Research Unit, University of Liège, Chemin de la vallée 4, Liège 4000, Belgium; Genetics and Physiology of Microalgae, InBios/Phytosystems Research Unit, University of Liège, Chemin de la vallée 4, Liège 4000, Belgium; de Duve Institute and MASSPROT platform, UCLouvain, Avenue Hippocrate 74, Brussels 1200, Belgium; de Duve Institute and MASSPROT platform, UCLouvain, Avenue Hippocrate 74, Brussels 1200, Belgium; Genetics and Physiology of Microalgae, InBios/Phytosystems Research Unit, University of Liège, Chemin de la vallée 4, Liège 4000, Belgium; VIB-VUB Center for Structural Biology, Pleinlaan 2, Brussels 1050, Belgium; Brussels Center for Redox Biology, Pleinlaan 2, Brussels 1050, Belgium; Structural Biology Brussels, Vrije Universiteit Brussel, Pleinlaan 2, Brussels 1050, Belgium; Genetics and Physiology of Microalgae, InBios/Phytosystems Research Unit, University of Liège, Chemin de la vallée 4, Liège 4000, Belgium

**Keywords:** APX2, *Chlamydomonas*, Copper deficiency, Cytochrome *c_6_*, P700, Plastocyanin

## Abstract

The function of ascorbate peroxidase–related (APX-R) proteins, present in all green photosynthetic eukaryotes, remains unclear. This study focuses on APX-R from *Chlamydomonas reinhardtii*, namely, ascorbate peroxidase 2 (APX2). We showed that *apx2* mutants exhibited a faster oxidation of the photosystem I primary electron donor, P700, upon sudden light increase and a slower re-reduction rate compared to the wild type, pointing to a limitation of plastocyanin. Spectroscopic, proteomic and immunoblot analyses confirmed that the phenotype was a result of lower levels of plastocyanin in the *apx2* mutants. The redox state of P700 did not differ between wild type and *apx2* mutants when the loss of function in plastocyanin was nutritionally complemented by growing *apx2* mutants under copper deficiency. In this case, cytochrome *c_6_* functionally replaces plastocyanin, confirming that lower levels of plastocyanin were the primary defect caused by the absence of APX2. Overall, the results presented here shed light on an unexpected regulation of plastocyanin level under copper-replete conditions, induced by APX2 in *Chlamydomonas*.

## Introduction

Ascorbate peroxidases (APXs) (EC 1.11.1.11) are heme *b*–containing enzymes belonging to class I peroxidases and catalyze the reduction of H_2_O_2_ to H_2_O using ascorbate as an electron donor. Several nucleus-encoded APXs are present in the different compartments of plants and algae (Caverzan et al. 2012). In chloroplasts, for example, APXs protect cells against oxidative damage (Shigeoka et al. 2002, Foyer and Noctor 2011). Their primary function is carried out on the stromal side of the chloroplasts, where the superoxide anion produced by photoreduction of O_2_ at the level of photosystem I (PSI) is converted into H_2_O_2_ by superoxide dismutase (Asada 1999).

Beside the classic APXs, a new class of APX-related (APX-R) enzymes was identified (Lazzarotto et al. 2021a). This class is present in all the green photosynthetic eukaryotes (Lazzarotto et al. 2015) and typically encoded by a single locus (Dunand et al. 2011). It is characterized by the absence of the amino acids typical for ascorbate binding, while the amino acids for heme binding and catalysis are conserved (Lazzarotto et al. 2021a). Most of the information regarding APX-Rs comes from studies in *Arabidopsis thaliana* (hereafter *Arabidopsis*), where the chloroplast localization of the APX-R enzyme (APX6) has been observed (Lazzarotto et al. 2021b). In vitro studies showed that APX6 does not use ascorbate to reduce H_2_O_2_ (Lazzarotto et al. 2021b). In vivo, *APX6* expression is increased during seed maturation, germination and leaf senescence (Chen et al. 2014, 2021) and responds to stresses, such as high light combined with high temperature (Giraud et al. 2008). In overexpressing transgenic plant lines, APX6 follows a degradative path from chloroplast plastoglobuli to cytoplasm during photomorphogenesis (Lazzarotto et al. 2021b). APX-R expression was also studied in a few other plants. In *Triticum aestivum*, expression of APX-R is down- or upregulated during drought, heat stress or their combination (Tyagi et al. 2020). In *Brassica rapa*, the expression of APX-R is differentially expressed according to stress exposure to drought and heat (Verma et al. 2022). In *Oryza sativa*, the expression is upregulated during drought or cold stress and in the presence of aluminum and UV light. In addition, mutant lines defective for APX-R display a growth delay (Lazzarotto et al. 2011).

Overall, no detailed information regarding the physiology and the photosynthetic activity of the abovementioned land plants has been provided so far.

Previous work from our groups recently showed that an APX-R of the green alga *Chlamydomonas*, namely, APX2, is chloroplast-localized and exhibits a twin-arginine translocation (TAT) motif for targeting the lumen of the thylakoids and a MxxM motif, typical for metal binding. In addition, the recombinant protein does not use ascorbate as an electron donor for reduction of H_2_O_2_, confirming its classification to the APX-R family. Additionally, it binds both copper and heme, and it modulates the copper-binding capabilities of plastocyanin in vitro (Caccamo et al. 2023).

With the aim of elucidating the link between APX2 and plastocyanin, we decided to investigate the physiology of *apx2* insertional mutants. Our detailed analysis of the photosynthetic machinery revealed that the mutants were not affected at the level of photosystem II (PSII). However, when transferred from low light to a sudden increase of light, *apx2* mutants exhibited a different redox state of P700, the PSI primary electron donor. This defect was caused by reduced levels of the photosynthetic electron carrier, plastocyanin. This phenotype was rescued under copper-deficient conditions, in which cytochrome *c_6_* functionally replaces plastocyanin, confirming that plastocyanin deficiency was the sole responsible factor for the mutant phenotype. Overall, these results provide support for the role of APX2 in regulating plastocyanin levels under copper-replete conditions in *Chlamydomonas*.

## Results

### The *apx2* mutants display undetectable levels of APX2

**Fig. 1:**
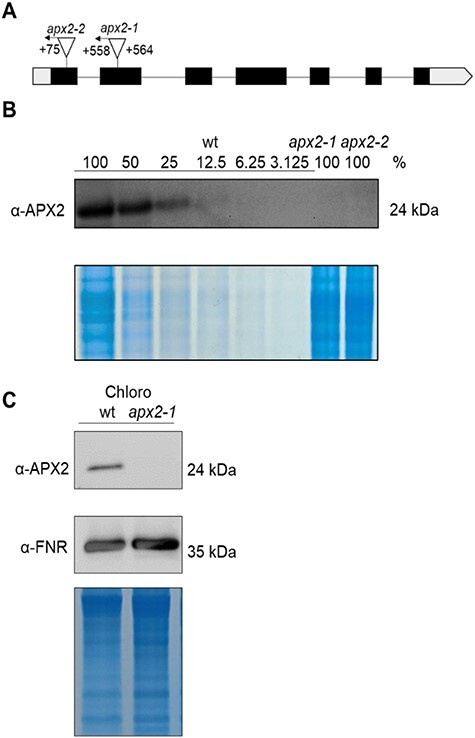
Mutants of *APX2* exhibit levels of the APX2 protein that are beyond detection. (A) Scheme of the *APX2* gene (Cre06.g285150) and position of the paromomycin cassette in the *apx2-1* and *apx2-2* mutants. The numbers indicate the position of the cassette from the ATG start codon. The arrows indicate the orientation of the cassette. Empty blocks are the 5ʹ UTR and the 3ʹ UTR, and filled blocks are the exons. (B) Immunoblot using soluble protein extracts with a serial dilution of a factor two for wt (100% is 30 μg of loaded extracts, 50% is 15 μg, 25% is 7.5 μg, 12.5% is 3.25 μg, 6.25% is 1.625 μg and 3.125% is 0.8125 μg) and the two *apx2* mutants (100% with 30 μg) labeled with antibodies against APX2. The Coomassie staining of the gel is shown as control of loading. (C) Immunoblot using chloroplast fractions (15 μg per lane) from wt and the *apx2-1* mutant labeled with antibodies against APX2 and FNR (Ferredoxin-NADP^+^ reductase) (shown as loading control) together with the Coomassie staining of the gel.

The *APX2* gene model (Cre06.g285150) as described in Phytozome (https://phytozome-next.jgi.doe.gov/) contains seven exons and six introns, has a length of 3,568 bp and codes for a protein of 337 amino acids (**Fig. 1A**). Two insertional mutants of the *APX2* gene, containing the mutagenesis cassette in exon 2 (*apx2-1*, LMJ.RY0402.180063) and exon 1 (*apx2-2*, LMJ.RY0402.095128), have been ordered from the Chlamydomonas Library Project (CLiP) library (**Fig. 1A**). To confirm the *APX2* gene disruption, we first checked the integration site of the cassette by amplifying the cassette junctions by PCR followed by sequencing of the amplified products (**Supplementary Table S1**). We were able to map both sides for the *apx2-1* mutant and only the 3ʹ side for the *apx2-2* mutant (**Fig. 1A**). The reason of failure for the *apx2-2* mutant could be the presence of a genomic rearrangement at the 5ʹ insertion site of the cassette (Li et al. 2016, 2019), which could prevent the sequencing of the flanking region. Polyclonal antibodies were raised against the purified recombinant protein expressed in *Escherichia coli*. Their sensitivity was assessed by a dilution series of the wild type (wt). For that purpose, 30 µg of wt-soluble extracts was diluted by a factor two (100%, 50, 25, 12.5, 6.25, 3.125%) and the immunoblot was labeled with the APX2 antibodies. A signal corresponding to the expected size of the mature form (24 kDa, Caccamo et al. 2023) was detected in wt-soluble extracts and absent in the *apx2* mutants. The detection limit was between 12.50 and 6.25%, indicating that the level of APX2 in the *apx2* mutants was below 12.5% of that of wt (**Fig. 1B**, **Supplementary Fig. S1A**). Since our previous work demonstrated that APX2 is chloroplast-localized and exhibits a TAT motif for targeting the lumen of the thylakoids (Caccamo et al. 2023), chloroplast fractions of the wt and of one of the *apx2* mutants (the *apx2-1* allele) were isolated and analyzed on immunoblot (**Fig. 1C**, **Supplementary Fig. S1B**). The 24-kDa band was not detected in the chloroplasts of the *apx2-1* mutant, while it was clearly visible in the chloroplast fraction of wt.

### *apx2* mutants display normal growth and PSII activity under low light but with a potential PSI activity defect

To evaluate the impact of the loss of APX2 on the physiology of *Chlamydomonas*, the growth rates of the *apx2* mutants were determined in phototrophy (minimal medium, low light intensity) and mixotrophy (acetate as organic carbon source, low light intensity). The growth rates of the mutants were similar to those of wt cells except for a minor difference for the *apx2-2* mutant in mixotrophy (**Table 1**). These results suggest that the loss of APX2 is not responsible for physiological modifications that affect growth under low light conditions.

**Table 1:** The growth rate of both wt and *apx2* mutants is comparable

Cells	Phototrophy Low light	Mixotrophy Low light
wt	37 ± 4	8 ± 1
*apx2-1*	38 ± 5	9 ± 1
*apx2-2*	34 ± 4	7 ± 1*

Doubling time (h) of wt and *apx2* mutant cells grown under low light (30 μmol photons m^−2^ s^−1^) in phototrophy and mixotrophy is shown. Values are means of six independent biological replicates with standard deviations. Statistical differences were calculated using one-way ANOVA followed by Dunnett’s test, * *P* < 0.05.

**Fig. 2:**
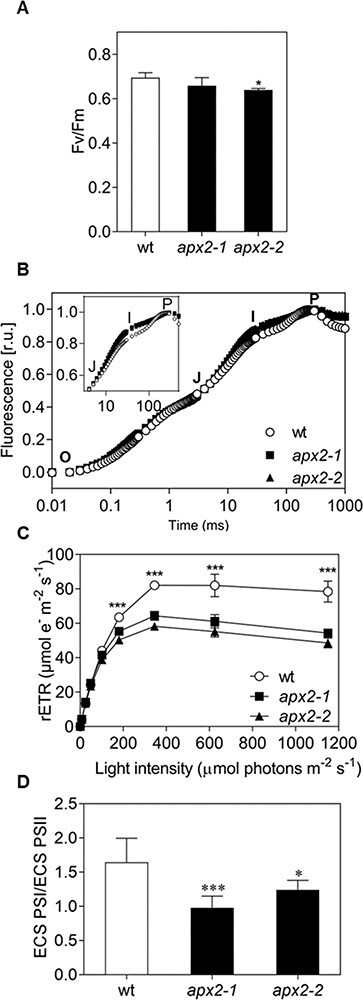
*apx2* mutants display no defect in PSII activity under low light but a possible defect around PSI. (A) Maximum quantum yield of PSII (Fv/Fm) measured after 1 min of dark incubation (*n* = 6). (B) Fast chlorophyll *a* fluorescence increase was measured during 1 s of saturating light after 1 min of dark cell incubation [r.u.: relative units of normalized fluorescence between O (20 µs) and P levels]. Inset in (B): Zoom on the increase of the J to the intermediate I phase for the *apx2* mutants (*n* = 6). (C) Light-response curve of photosynthetic rETR at increasing light intensities with measurements of 30 s per each light step (*n* = 3). (D) Ratio between active PSI and PSII centers calculated from ECS measurements in the presence of PSII inhibitors (*n* = 9). Values are shown as averages with standard deviations. Statistical differences were calculated using one-way ANOVA for (A) and (D), or two-way ANOVA for (C) followed by Dunnett’s test, *** *P* < 0.001, * *P* < 0.05.

Next, we evaluated the photosynthetic activity. In order to maximize the use of photosynthesis, the cells were grown under phototrophic conditions. Under these cultivation conditions, the maximum efficiency of PSII was not affected, except for a small decrease (8%) for the *apx2-2* mutant (**Fig. 2A**). The initial phases (O–J, **Fig. 2B**) of the rapid rise in chlorophyll *a* fluorescence in PSII, associated with photochemistry (Kitajima and Butler 1975), showed similar trends. Additionally, the relative electron transport rate (rETR) of PSII remained unaffected at low light intensities (**Fig. 2C**). All these results indicated that the PSII activity in low light is barely affected in the *apx2* mutants, aligning with the calculated growth rates (**Table 1**).

Nevertheless, the decline in rETR of PSII under high light conditions (i.e. beyond 100 µmol photons m^−2^ s^−1^, **Fig. 2C**) was observed in the *apx2* mutants. Besides, the faster increase in the J–I phase during the analysis of the rapid rise in chlorophyll *a* fluorescence suggested a donor site limitation of PSI (Zivcak et al. 2015) (**Fig. 2B**, inset). Lastly, the ratio between the active PSI and PSII centers (PSI/PSII) was lower in the mutants, indicating a potential deficiency of PSI (**Fig. 2D**).

### Reduced levels of plastocyanin in *apx2* mutants lead to rapid oxidation of P700

**Fig. 3:**
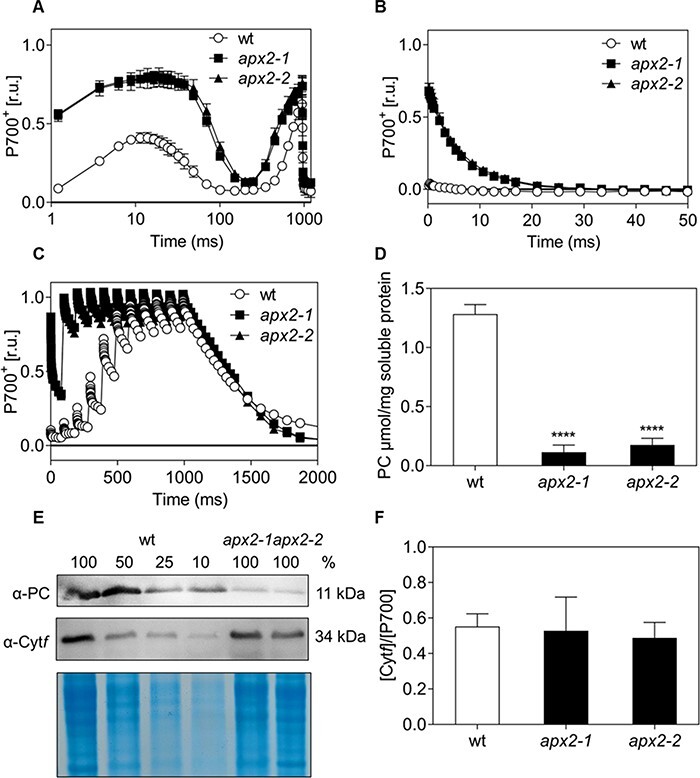
The electron transfer to P700 was impaired in the *apx2* mutant cells, accompanied by a reduced level of plastocyanin. (A) P700 redox kinetics measured during 1 s of saturating light after 1 min of dark incubation. Values are normalized to maximum oxidizable P700 in the presence of DCMU and DBMIB, inhibitors of PSII and Cyt *b*_6_*f*, respectively (*n* = 3). (B) Monitoring of the P700 redox state upon a single-turnover flash on wt and *apx2* mutants (values normalized to the maximum oxidizable value of P700) (*n* = 3). (C) Monitoring of the P700 redox state during a train of 10 single-turnover flashes (every 100 ms) in wt and *apx2* mutants previously incubated in PSII and Cyt *b*_6_*f* inhibitors (values normalized to the maximum oxidizable value of P700 after adding 10 μM DCMU and 10 μM DBMIB inhibitors of PSII and Cyt *b*_6_*f*, respectively) (*n* = 3). (D) Plastocyanin (PC) intracellular level in wt and *apx2* mutants measured using spectroscopic analyses (*n* = 3). (E) Immunoblot with a serial dilution for wt (100% is 30 μg of loaded extracts, 50% is 15 μg, 25% is 7.5 μg and 10% is 3 μg) labeled with antibodies against plastocyanin (PC) and Cyt*f*. Coomassie blue–stained gel with the same loadings is shown as control. (F) Ratio between [Cyt*f*] and [P700] (changes in absorption values calculated considering the respective molar extinction coefficients of 18 mM^−1^ cm^−1^ at 554 nm for Cyt*f* and of 50 mM^−1^ cm^−1^ at 705 nm for P700) (*n* = 4). Statistical differences were calculated with one-way ANOVA followed by Dunnett’s test for (D) and (F), **** *P* < 0.0001.

To further investigate PSI activity, we monitored P700 oxidation during 1 s of saturating light (**Fig. 3A**). *apx2* mutants exhibited a more oxidized P700 redox state, during the first 100 ms of the saturating light intensity, indicating a donor side limitation of PSI. To determine the underlying cause of the observed effect in the mutant cells, we conducted a single-turnover flash experiment to induce a single charge separation in active photosystems. It is worth mentioning that the electron transfer between plastocyanin and P700^+^ occurs rapidly, with time constants of approximately 1–3 μs (Drepper et al. 1996). Considering this fast electron transfer, the first detection in the P700 monitoring system (200 μs after the single-turnover flash) could not detect the oxidized state in the wt (**Fig. 3B**), but it was surprising to see that *apx2* mutant cells retained the oxidized state for a longer period with a constant time for P700^+^ re-reduction of approximately 1 ms (50% of P700^+^ re-reduced). Such a difference suggested an impairment at the donor side of PSI.

To assess the size of the PSI electron donor pool (i.e. mainly plastocyanin), the P700 redox state was monitored in vivo in response to a train of 10 consecutive single-turnover flashes and in the presence of inhibitors of PSII and Cyt *b*_6_*f*. In wt, the oxidation level of P700 was increased with consecutive flashes, indicating a progressive withdrawal of electrons from the plastocyanin pool to PSI. The maximum P700 oxidation level was obtained after seven flashes. In contrast, *apx2* mutant cells required only two flashes to reach the maximum P700 oxidation level (**Fig. 3C**). This result suggests a significant reduction in the size of the plastocyanin pool in the absence of APX2. Spectrophotometric quantification of plastocyanin on soluble protein extracts demonstrated a substantial decrease in plastocyanin content in the *apx2* mutants compared to wt cells (**Fig. 3D**). Lower abundance was further confirmed by immunoblots labeled with plastocyanin antibodies, showing that the quantity of plastocyanin in the *apx2* mutants is less than 10% of that in wt cells (**Fig. 3E**, **Supplementary Fig. S1C**). In contrast, the molar stoichiometry of Cyt*f*^+^ to P700^+^ remained consistent between wt and the *apx2* mutants (**Fig. 3F**) as well as the level of Cyt*f*, as indicated by an immunoblot analysis (**Fig. 3E**).

### Proteomic analyses of the *apx2-1* mutant validate the reduced abundance of plastocyanin and uncover deficiencies in PSI subunits, as well as copper-related enzymes

**Fig. 4:**
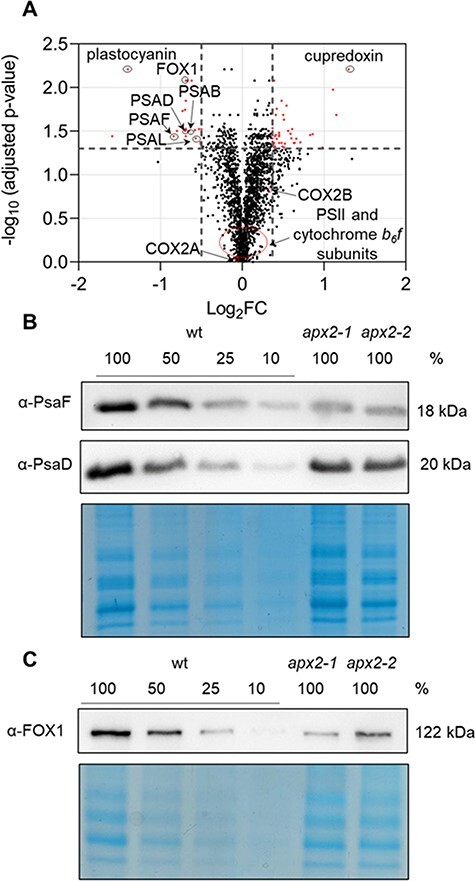
Comparative proteomic analysis reveals variations in protein levels in the *apx2-1* mutant, which are confirmed by immunoblot in both mutants. (A) Volcano plots of the proteomic data. Red dots indicate significantly different proteins in the *apx2-1* mutant compared to wt (log2 fold change > 0.37 and < −0.5). Black dots indicate the non-significant proteins. In the plot plastocyanin, PSI subunits (PSAB, PSAD, PSAF and PSAL), copper enzymes (FOX1, cupredoxin) and subunits of the cytochrome *c* oxidase (COX2A and COX2B) are indicated. The PSII and cytochrome b_6_*f* subunits are shown in the non-significant portion of the plot. (B) Immunoblot with a serial dilution for wt (100% is 30 μg of loaded extracts, 50% is 15 μg, 25% is 7.5 μg and 10% is 3 μg) labeled with antibodies against PSAF and PSAD. Coomassie blue–stained gel with the same loadings is shown as control. (C) Immunoblot with a serial dilution for wt (100% is 30 μg of loaded extracts, 50% is 15 μg, 25% is 7.5 μg and 10% is 3 μg) labeled with antibodies against FOX1. Coomassie blue–stained gel with the same loadings is shown as control.

Since plastocyanin represents the major sink of copper in *Chlamydomonas* under phototrophic conditions (Kropat et al. 2015), we wanted to investigate the whole proteome to identify any variation of copper-related proteins. For this reason, tandem-mass-tag (TMT) labeling (Zecha et al. 2019) was used to semi-quantitatively analyze the whole cell proteome of *Chlamydomonas* in phototrophy for the wt and the *apx2-1* mutant only, since the two *apx2* mutants showed the same phenotype. Using this technique, we could identify and quantify 2,502 and 2,255 proteins, respectively (Dataset 1, **Supplementary material**). Variations in abundance were considered significant when there was an increase or a decrease of 30% compared to the wt, with an adjusted *P* value of <0.05 (log_2_ fold change ≥0.37 and ≤−0.5) (Dataset 1, **Supplementary material**, **Fig. 4A**). Twenty-one proteins were found to be significantly decreased, and 42 were significantly increased. As expected, there was a notable decrease in the quantity of plastocyanin (log_2_ fold change −1.4). We successfully identified six subunits of PSI, six subunits of cytochrome *b*_6_*f* and nine subunits of PSII (Dataset 1, **Supplementary material**). No significant differences were observed for these proteins except for some subunits of PSI. Indeed, four PSI core subunits (i.e. PsaB, PsaD, PsaF and PsaL) exhibited a significant decrease in abundance. These findings were validated through immunoblot analysis using antibodies specifically targeting PsaF and PsaD subunits (**Fig. 4B**, **Supplementary Fig. S1D**). These results support the finding of a reduced PSI/PSII ratio and the absence of any PSII defects (**Fig. 2**) in these *apx2* mutants.

Regarding other copper-related proteins beyond plastocyanin, we did observe a significant difference in abundance for two of them, ferroxidase-like 1 (FOX1) and a cupredoxin multicopper enzyme (Cre12.g537250). FOX1, reported as a multicopper periplasmic oxidase involved in iron uptake (Terzulli and Kosman 2010), showed a log_2_ fold change of −0.7 in the absence of APX2, whereas Cre12.g537250 was found to be more abundant in *apx2-1* mutant cells (log_2_ fold change 1.3) (**Fig. 4A**, Dataset 1, **Supplementary material**). The higher abundance of this enzyme in the *apx2-1* mutant might suggests a function as a copper storage. However, no information is available about Cre12.g537250 and its localization, and the hypothesis on its function in this context remains speculative. The reduction in FOX1 protein was verified through immunoblot analysis for the two *apx2* mutants (**Fig. 4C**, **Supplementary Fig. S1E**). On the contrary, there was no observed alteration in protein levels of several other copper proteins: copper transporters CTR1 and CTR3, copper chaperone ATX1 and one of the copper-binding subunits of the mitochondrial cytochrome *c* oxidase (COX2B) (**Fig. 4A**, Dataset 1, **Supplementary material**). Altogether, these findings suggest that the pattern of copper metabolism could be modified in the mutants, although not to a significant extent.

### Copper deficiency induces a similar response in wt and *apx2* mutants

**Fig. 5:**
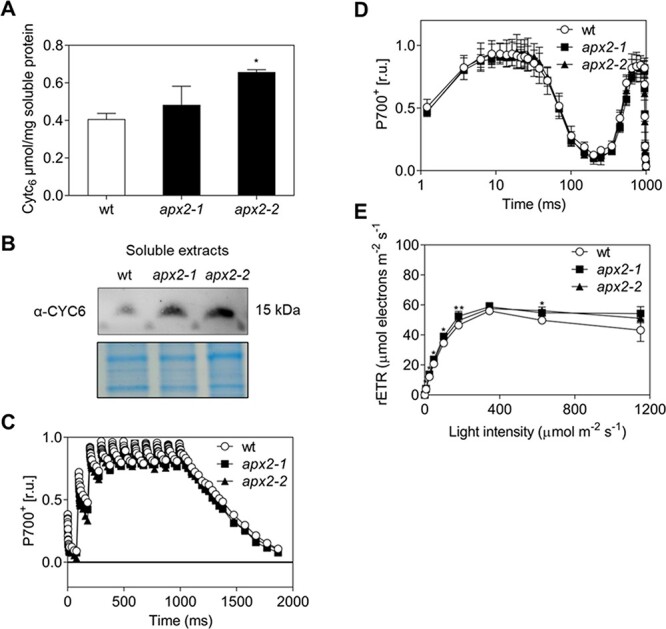
Induction of cytochrome *c*_6_ under copper deficiency restores a wt phenotype in *apx2* mutants. (A) Cytochrome *c*_6_ (Cyt*c*_6_) intracellular level in wt and *apx2* mutants measured using spectroscopic analyses (*n* = 3). (B) Immunoblot labeled with cytochrome *c*_6_ (CYC6) antibodies of soluble extracts (10 μg per lane) separated by Tricine gel in the presence of 1 nM CuSO_4._ The lower panel shows a gel stained with Coomassie blue for loading control. (C) wt and *apx2* mutant cells exposed to copper deficiency were subjected to a train of 10 consecutive single-turnover flashes (*n* = 3). (D) P700 redox kinetics measured during 1 s of saturating light after 1 min of dark incubation. Values are normalized to maximum oxidizable P700 in the presence of DCMU and DBMIB, inhibitors of PSII and Cyt *b*_6_*f*, respectively (*n* = 3). (E) Light-response curve of photosynthetic rETR at increasing light intensities with measurements of 30 s per each light step (*n* = 3). Significant differences between *apx2-1* and wt are present for several light steps: 7 µmol photons m^−2^ s^−1^: +9%; 25 µmol photons m^−2^ s^−1^: +13%; 48 µmol photons m^−2^ s^−1^: +14%; 101 µmol photons m^−2^ s^−1^: +12%; *apx2-2* and wt at 101 µmol photons m^−2^ s^−1^: +7%; 180 µmol photons m^−2^ s^−1^: +7% and 625 µmol photons m^−2^ s^−1^: +13%. Values are means with standard deviations calculated using ANOVA one-way, Dunnett’s test: values indicated by ** equal *P* < 0.01, values indicated by * equal *P* < 0.05 and nothing equals *P* > 0.05, relative to wt, for (A and E).

To investigate further the impact of *APX2* gene disruption at the level of the PSI electron donor, we decided to evaluate the functional replacement of plastocyanin by cytochrome *c*_6_ induced by copper deficiency (Li and Merchant 1995). Previously, it has been demonstrated that *Chlamydomonas* cells utilize cytochrome *c*_6_ (Cre16.g651050) as a PSI electron donor below concentrations of 20 nM of copper in the culture medium (Hill and Merchant 1992). Therefore, cells were grown under phototrophic conditions in the presence of 1 nM CuSO_4_. We observed that after 10 days of copper deficiency, cytochrome *c*_6_ took over the role of plastocyanin, as evidenced by the quantification of its characteristic absorption peak at 552 nm in soluble protein extracts (**Fig. 5A**) and confirmed by immunodetection using antibodies specific to cytochrome *c*_6_ (**Fig. 5B**, **Supplementary Fig. S1F**). Cytochrome *c*_6_ seems to be more abundant in the mutants. Further experiments will be required to understand the mechanisms underlying this increased level. Interestingly, under these conditions, the oxidation level of P700 induced by a series of 10 consecutive single-turnover flashes displayed identical kinetics in both the wt and *apx2* mutant cells (**Fig. 5C**). Additionally, we do not observe any changes in the P700 kinetics between wt and *apx2* mutants (**Fig. 5D**). These observations indicate that the pool of electron donors to PSI becomes comparable between the mutants and the wt cells under copper-limited conditions. This would also suggest that the levels of the PsaF and PsaD subunits are also comparable between the wt and the *apx2* mutants under copper-limited conditions, although this hypothesis was not verified. In addition, even if variations in the rETR are observed (between 7 and 14%; **Fig. 5E**), PSII activity is restored even upon high light intensities, contrary to what is observed in the presence of copper (**Fig. 2C**). We concluded that the absence of APX2 adversely affects plastocyanin, without compromising the ability to substitute plastocyanin with cytochrome *c*_6_ during copper deficiency.

### Transcriptomics analyses reveal post-transcriptional regulation under copper-replete conditions and no modification of the copper response regulator 1 targets under copper-deficient conditions

From the aforementioned results, it can be concluded that the main differences between wt and *apx2* mutants are present under copper-replete conditions and not under copper-deficient conditions. To further understand the cellular regulation, a transcriptomics study was undertaken by comparing *apx2-1* and wt cells grown in phototrophy under both copper-replete (6 µM CuSO_4_) and copper-deficient (1 nM CuSO_4_) conditions. To achieve this, RNA was extracted from three biological replicates for each cultivation condition and RNA-seq data were acquired. After quality check, approximately 20 million reads could be uniquely mapped, indicating the high quality of the libraries (Dataset 2, **Supplementary material**). The generated raw data are available under the project number PRJNA994826.

Differentially expressed genes (adjusted *P* value < 0.05 and log_2_ fold change > or <1) were identified in wt and *apx2-1* grown in copper-replete medium (6 µM CuSO_4_) (wt-apx2) and in wt [wt–wt (1 nM Cu)] and *apx2-1* [apx2–apx2 (1 nM Cu)] grown in copper-deficient medium (1 nM CuSO_4_) (Dataset 2, **Supplementary material**). **Supplementary Fig. S2A** presents a heat map of the most upregulated and the most downregulated genes in each comparison. Many of these genes encode uncharacterized proteins.

**Fig. 6:**
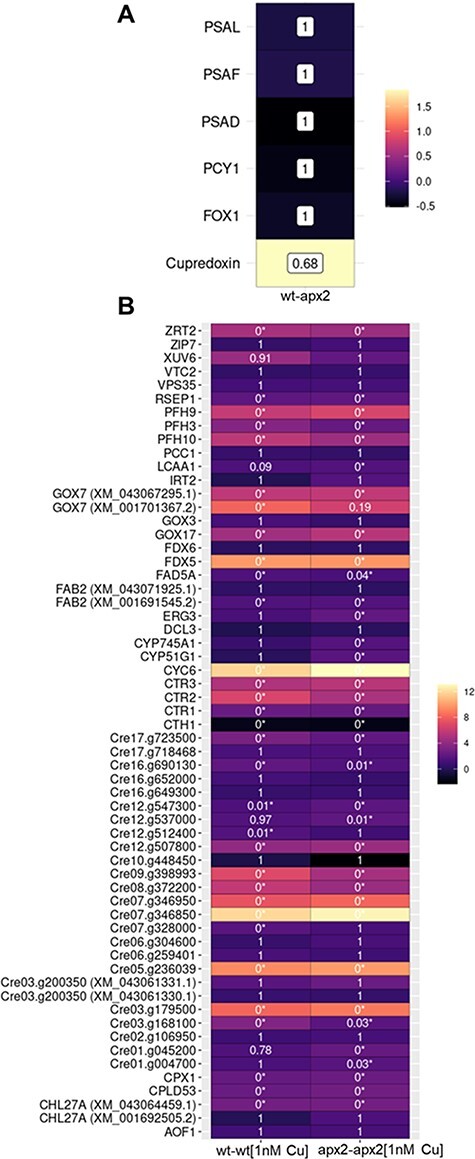
Transcriptomics analyses reveal post-transcriptional differences between wt and the *apx2-1* mutant under copper-replete conditions and similar response to CRR1 master regulator under copper-deficient conditions. (A) Expression of *PCY1, PsaF, PsaD, PsaL, FOX1* and *cupredoxin* is not significantly different between wt and *apx2-1* mutant. The color code at the right shows the difference in log_2_ fold change. The adjusted *P* value is shown in the boxes corresponding to each comparison. (B) Comparison between wt and *apx2-1* mutant in response to copper deficiency. The color code at the right shows the difference in log_2_ fold change. The adjusted *P* value is shown in the boxes corresponding to each comparison with significantly differential expressed genes indicated with asterisks.

Regarding the comparison between wt and *apx2-1* in copper-replete medium (wt-apx2), the transcripts encoding those proteins with altered abundance (plastocyanin, PSAD, PSAF, PSAL, FOX1, cupredoxin, **Fig. 4A**) are not differentially expressed, suggesting that the regulation occurs at the post-transcriptional level (**Fig. 6A**).

In copper deficiency, both the wt [wt–wt (1 nM Cu)] and the *apx2*-1 mutant [apx2–apx2 (1 nM Cu)] display higher abundance of some transcripts governed by the copper response regulator 1 (CRR1) transcription factor (Cre09.g390023), such as Cyt *c*_6_ and FDX5-encoding ferredoxin 5 (**Supplementary Fig. S2A**). This prompted us to look at the abundance of the 58 target genes of CRR1 (Kropat et al. 2015). Out of these targets, one gene was excluded from our analysis (Cre07.g346900) due to insufficient mapped reads. The remaining 57 genes showed consistent differential expression in both strains (**Fig. 6B**). However, for some of them, the log_2_ fold change was not significant, in contrast to previous observations by Kropat et al. (2015). This discrepancy is likely attributed to variations in growth conditions used in the two studies. In conclusion, even though there are differences between wt and *apx2* mutants in copper deficiency, they do not seem to be linked to the CRR1 targets and will be complicated to interpret due to the absence of a phenotype in this condition.

## Discussion

APX-R enzymes form a recently identified class within the APX family, present in the eukaryotic green lineage (Lazzarotto et al. 2015). Previous studies on loss-of-function mutant lines in *Arabidopsis* have suggested that the APX-R enzyme plays a role during stress periods, such as seed maturation, germination and leaf senescence (Chen et al. 2014, 2021), although the precise underlying mechanisms remain unknown. Despite the chloroplast localization of APX-R, no interaction with the photosynthetic machinery has been explored in any photosynthetic organism.

Our previous in vitro work demonstrated that recombinant APX2 binds copper and that copper-loaded APX2 interferes with the copper-binding capabilities of plastocyanin (Caccamo et al. 2023). The detailed photosynthetic analysis performed in this study reveals that the main defect of the *apx2* mutants is a significant reduction of their plastocyanin levels. Therefore, the relation between plastocyanin and APX2 is confirmed in vivo in *Chlamydomonas*.

The diminished plastocyanin levels in the *apx2* mutants raise the question of how the electron transport can still take place. In *Arabidopsis*, there are two genes encoding plastocyanin, *PETE1* and *PETE2* (Abdel-Ghany 2009). While PETE2 is the predominant isoform, PETE1 is less abundant and substitutes PETE2 in copper deficiency (Abdel-Ghany 2009). Mutants of *pete1* and *pete2* exhibit reduced plastocyanin content, and null *pete2* mutants, with 90% reduction of plastocyanin content, only show a slight effect on growth and photosynthesis (Pesaresi et al. 2009). Double mutants are not viable in *Arabidopsis* (Weigel et al. 2003). Consequently, a low level of plastocyanin is not limiting under optimal growth conditions, being under day/night cycles, suggesting another role for the protein that remains to be deciphered (Pesaresi et al. 2009). Similarly, based on the spectroscopic and immunoblot analyses, the results presented here show that a plastocyanin level less than 10–15% of the wt does not affect the growth under low light. Considering the effect that the lack of APX2 exerts on the plastocyanin content, a question about the nature of unveiled interactors of plastocyanin in *Chlamydomonas* arises.

Apart from *Arabidopsis*, other land plants such as poplar, parsley, tobacco, rice and the moss *Physcomitrella patens* express two plastocyanin isoforms (as described in Pesaresi et al. 2009). In contrast, green algae and cyanobacteria, such as *Chlamydomonas* and *Synechocystis*, respectively, possess only one plastocyanin gene (Merchant and Bogorad 1987, Briggs et al. 1990, Pesaresi et al. 2009). This raises the question of whether APX-R of *Arabidopsis* (APX6) would have a similar impact on plastocyanin levels.

The TAT signal motif for lumen translocation consists of a twin arginine (RR) followed by a hydrophobic stretch and an AxA cleavage site (Cline 2015). When comparing *Chlamydomonas* and *Arabidopsis* APX-R sequences, we could notice the absence of the AxA cleavage site in APX6 (**Supplementary Fig. S3)**. This suggests that APX6, despite being chloroplast-localized (Lazzarotto et al. 2021b), might be not directed to the lumen. In addition, *APX6* is transcribed under copper-replete condition, while in copper deficiency, its transcription is repressed by miR398, which is regulated by SQUAMOSA-promoter-binding protein-like 7 (SPL7), an ortholog of the CRR1 of *Chlamydomonas* (Chen et al. 2021). In *Chlamydomonas*, the transcriptomics analysis presented here shows that *APX2* is transcribed under both copper-replete and copper-deficient conditions in wt (Dataset 2, **Supplementary material**, **Supplementary Fig. S2B**). Thus, the function of the APX-R might differ between the green algae and the land plants. Nevertheless, both APX-R contain the MxxM motif, typical for metal binding (Caccamo et al. 2023) (**Supplementary Fig. S3**), suggesting that their dependence on copper could be conserved.

In conclusion, this study demonstrates that APX2 regulates the plastocyanin level under a standard copper concentration in *Chlamydomonas*. This discovery paves the way for future experiments that could unveil the yet undiscovered mechanisms underlying the relation between APX2 and plastocyanin and, more broadly, the biogenesis of this photosynthetic component.

## Material and Methods

### Strains and culture media

wt (CC-4533 *cw15* mt-) and *apx2* mutants of *Chlamydomonas* (*APX2* Cre06g.285150) have been ordered from the *Chlamydomonas* library (https://www.chlamylibrary.org/) (Li et al. 2019). We will refer to them as wt, *apx2-1* (LMJ.RY0402.180063) and *apx2-2* (LMJ.RY0402.095128). wt and mutant strains were grown at 25°C under a phototrophic minimal condition in Tris-minimal-phosphate (TMP) medium or under a mixotrophic condition in Tris-acetate phosphate medium in standard copper concentration (6 µM CuSO_4_) (Hutner et al. 1950) or under copper-low conditions [TMP + 1 nM CuSO_4_], under continuous low light (30 µmol photons m^−2^ s^−1^) and with gentle shaking (Harris 2009).

### Growth measurements

Algal growth was determined two times per day by measuring the optical density (OD) at 750 nm until the stationary phase was reached, using a 265 UV/Vis spectrophotometer (LAMBDA^TM^ 265, PerkinElmer, Belgium). The growth rate *μ* (*h* − 1) of the cell cultures was calculated as in (Jia et al. 2015), *μ* (*h* − 1) = [ln(OD2) − ln(OD1)]/(*t*_2_ − *t*_1_), in which *μ* is the growth rate (*h* − 1), OD is the optical density of cells at the end (OD2) and beginning (OD1) of the exponential phase, *t*_2_ is the time in h at the end of the exponential phase and *t*_1_is the time in h at the beginning of the exponential phase.

The doubling time was calculated as *t*_d_ = ln(2)/*μ*(*h* − 1).

### Total DNA extraction and PCR amplification

DNA extraction was performed by following the protocol described in Newman et al. (1990). The DNA was used for PCR amplifications to confirm the presence of the paromomycin cassette in *apx2* mutants. PCR amplifications were performed as suggested on the CLiP library website, using the couple of primers *apx2-1*_F/OMJ944 and *apx2-1*_R/OMJ913 to map the 3ʹ end and the 5ʹ end of the cassette, respectively, of the *apx2-1* mutant and primers *apx2-2*_F/OMJ944 to map the 3ʹ side of the cassette of the *apx2-2* mutant. Primer sequences are presented in **Supplementary Table S2**. Sequencing was made at Eurofins genomics.

### Total RNA extraction

Cultures of wt and *apx2-1* mutant were grown in triplicates in TMP and TMP + 1 nM CuSO_4_ under continuous low light at 30 µmol m^−2^ s^−1^. Cultures of TMP + 1 nM CuSO_4_ were started by washing the pellets of the pre-cultures grown in TMP-CuSO_4_.The pellets were suspended in TMP-CuSO_4_ and the cultures launched in TMP + 1 nM CuSO_4_. After 4 d, the cultures were refreshed with new TMP + 1 nM CuSO_4_ medium until plastocyanin was completely substituted by cytochrome *c_6_*. The cultures were then collected at the mid-exponential phase, the pellets were resuspended in 0.5 ml of 2% sodium dodecyl sulfate (SDS), 400 mM NaCl, 40 mM EDTA, 100 mM Tris/HCl, pH 8.0 and dimethyl pyrocarbonate to lyse the cells and total RNA was extracted using the NucleoSpin® RNA Plant and Fungi kit as described in the Macherey-Nagel protocol (Ref. 740120.50). The concentration and the purity (260/280 ratio 2.0–2.1) of RNA were determined using NanoDrop (Agilent BioTek Synergy Mx Monochromator-Based Multi-Mode Reader with Time-resolved Fluorescence) and using gel electrophoresis.

### Transcriptomic analyses

#### Sample collection and preparation.

*Chlamydomonas* wt and apx2-1 cells were grown in triplicate under the two cultivation conditions (12 samples) and collected for RNA extraction as described in the Total RNA extraction section. The samples were sent for sequencing to the Novogene Company (Novogene Company Limited, Cambridge, UK). Raw data are available at the National Center for Biotechnology Information with the BioProject ID number PRJNA994826.

#### RNA quantification and quality assessment (Novogene company).

RNA integrity was assessed using the RNA Nano 6000 Assay Kit of the Bioanalyzer 2100 system (Agilent Technologies, Santa Clara, California, USA).

#### Library preparation for transcriptome sequencing (Novogene experimental department).

Total RNA was used as input material for the library preparations, which was carried out by using Novogene NGS RNA Library Prep Set (PT042) according to the manufacturer’s protocol. In order to select cDNA fragments of preferentially 370–420 bp in length, the library fragments were purified using an AMPure XP system (Beckman Coulter, Beverly, Massachusetts, USA). Then, PCR was performed with Phusion High-Fidelity DNA polymerase, Universal PCR primers and Index (X) Primer. At last, PCR products were purified (AMPure XP system) and library quality was assessed on the Agilent Bioanalyzer 2100 system.

#### Clustering and sequencing (Novogene experimental department).

The clustering of the index-coded samples was performed on a cBot Cluster Generation System using TruSeq PE Cluster Kit v3-cBot-HS (Illumina, San Diego, California, USA) according to the manufacturer’s instructions. After cluster generation, the library preparations were sequenced on an Illumina Novaseq platform and 150 bp paired-end reads were generated.

#### Transcriptomic analyses.

Reads were quality trimmed using Trimmomatic software (Bolger et al. 2014) removing adapters and bases with the average score lower than 30 over 10 bp sliding window. Afterward, they were aligned and counted using Trinity software (Grabherr et al. 2011). *Chlamydomonas reinhardtii* v5.5 transcriptome data were downloaded from Phytozome (Goodstein et al. 2012) and indexed with bowtie2 (Langmead and Salzberg 2012). Transcripts, including isoforms and abundances, were obtained using RNA-Seq by expectation maximization (Li and Dewey 2011). The sequencing depth is presented in Dataset 2, **Supplementary material** (section Mapped_reads). The normalization and differential expression analysis were performed using DESeq2 R package (Love et al. 2014). The reported differentially expressed (DE) transcripts were obtained by selecting the results with log_2_FC > 1 or log_2_FC < −1 and false discovery rate (FDR)–adjusted *P* values ≤0.05.

### Chlorophyll content

Pigments were extracted from whole cells using cold pure methanol, following a protocol adapted from Emonds-Alt et al. (2017). Chlorophyll a and b concentrations were determined by measuring the absorbance at 652, 665 and 750 nm following (1) and (2) (Ritchie 2006):


(1) $${\mathrm{Chl}}\;a\left( {{\mu g/ml}} \right){\mathrm{: }} - {\mathrm{8}}{\mathrm{.0962*}}\left( {{\mathrm{Abs652}} - {\mathrm{Abs750}}} \right){\mathrm{ + 16}}{\mathrm{.5169*}}\left( {{\mathrm{Abs665}} - {\mathrm{Abs750}}} \right)$$

(2) $${\mathrm{Chl }}b\left( {{\mu g/ml}} \right){\mathrm{: 27}}{\mathrm{.4405*}}\left( {{\mathrm{Abs652}} - {\mathrm{Abs750}}} \right){\mathrm{ - 12}}{\mathrm{.1688*}}\left( {{\mathrm{Abs665}} - {\mathrm{Abs750}}} \right)$$

### Isolation of soluble and chloroplast-enriched fractions

Soluble fractions were prepared as in Li and Merchant (1992). Cultures of wt and *apx2* mutants were harvested in the mid-exponential phase and washed with sodium phosphate buffer 10 mM pH 7.0, 2000* g* for 3 min at room temperature. The pellet was suspended in the same buffer at 2 × 10^8^ cells ml^−1^ and frozen at −80°C. Five cycles of freeze–thaw for 1 h each were performed to extract soluble proteins. After the last cycle, the cells were centrifuged for 15 min at 4°C at 12,000 *g*, the supernatant and the pellet were collected and the protein content was determined using a Bradford assay, as in Ernst and Zor (2010). Chloroplast-enriched fractions were isolated according to Mason et al. (2006).

### Efficiency of PSII

In vivo chlorophyll *a* fluorescence was measured in 1-cm microcuvettes by using a Joliot-Type-Spectrophotometer (JTS-10, BioLogic, Seyssinet-Pariset, France). A blue light-emitting diode probe of 10 μs pulses was used to measure the chlorophyll *a* fluorescence under dark conditions (Fo) and after a saturating pulse of red light (Fm, 3000 µmol photons m^−2^ s^−1^) to reach the maximum PSII quantum yield (Fv/Fm = Fm − Fo/Fm). Samples were then acclimated to different photosynthetic photon flux densities (PPFD) for 3 min, and fluorescence values were measured (Fs). A saturating pulse was then given to fully reduce PSII acceptors and reach the maximal fluorescence yield (Fm'). These parameters were used to calculate the PSII quantum yield (ΦPSII = (Fm' − Fs)/Fm') and the relative electron transfer rate (rETR = ΦPSII × PPFD) (Maxwell and Johnson 2000).

### Fast fluorescence increase

The fast chlorophyll a fluorescence rises in response to a saturating pulse of light of 3,000 µmol photons m^−2^ s^−1^ and was obtained using a plant efficiency analyzer fluorometer (Handy PEA, Hansatech, Kings Lynn, UK) after a short dark incubation in 1-cm microcuvettes. The chlorophyll *a* fluorescence increases were normalized to variable fluorescence (i.e. between the values of Fo and Fm) (Strasser and Govindjee 1992).

### Activity of PSI

PSI activity was monitored using the Joliot-Type-Spectrophotometer (JTS-10, BioLogic France) by the changes in absorption in the near infra-red region of the P700, the primary electron donor at the PSI reaction center. The signal at 705 nm was corrected by the absorption at 725 nm. After a short period of dark incubation, a saturating red actinic light of 3000 µmol photons m^−2^ s^−1^ was given for 1 s. The measurements were normalized to the maximum change of absorption due to P700 oxidized in the presence of 10 µM of 3-(3,4-dichlorophenyl)-1,1-dimethylurea (DCMU) and 10 µM of 2,5-dibromo-3-methyl-6-isopropylbenzoquinone (DBMIB). The signal/level of oxidized cytochrome *f* (Cyt*f^+^*) from the cytochrome *b_6_f* complex was deconvoluted from the signal at 554 nm and a baseline between 546 nm and 573 nm in the presence of 10 µM of DCMU and 10 µM DBMIB. The relative abundance of Cyt*f^+^* and P700^+^ (Cyt*f^+^/*P700^+^) was calculated from molar extinction coefficients of 18 mM^−1^ cm^−1^ at 554 nm for Cyt*f* (Pierre et al. 1995) and of 50 mM^−1^ cm^−1^ at 705 nm for P700 (Heinnickel et al. 2013). Electron transfer to P700^+^ was monitored after a single-turnover flash, provided by a Nd-YAG laser (Minilite II, Continuum) generating one charge separation per photosystem. A train of 10 consecutive single-turnover flashes, triggered every 100 ms, was used to oxidize P700 in the presence of DCMU and DBMIB (Drepper et al. 1996).

### Electrochromic shift assay

The electrochromic shift (ECS), used to monitor the electric field of the thylakoid membranes in response to photosynthetic activity, was measured using the JTS-10. Absorption changes were monitored at 520 nm and corrected by measurements at 546 nm. A baseline of absorption in darkness was made, and afterward, a single-turnover flash was applied. The measurements were repeated with the addition of 10 µM DCMU and 1 mM hydroxylamine, preventing charge separations at PSII. The change in absorption corresponding to PSII and PSI or only to PSI charge separations was used to calculate the relative abundance of active PSI and PSII (Bailleul et al. 2010).

### Plastocyanin and cytochrome c_6_ quantification

Soluble proteins were extracted as described in Li and Merchant (1992). Cultures of wt and *apx2* mutants were harvested in the mid-exponential phase and washed with sodium phosphate buffer 10 mM pH 7.0, 2000 *g* for 3 min at room temperature. Soluble extracts were prepared as described earlier. Each sample was split into two, one oxidized with 1 mM potassium ferricyanide and the second one reduced with 1 mM of ascorbic acid. Absorbance was recorded between 400 and 800 nm. The plastocyanin concentration was calculated using a molar extinction coefficient for the oxidized form of 4.9 mM^−1^ cm^−1^ at 597 nm (Sommer et al. 2002). The actual absorbances of plastocyanin at 597 nm were calculated by subtracting the absorption value of the reduced sample to the oxidized one (Li and Merchant 1995) and correcting the signal by removing that one at 700 nm.

The cytochrome *c_6_* concentration was calculated using a molar extinction coefficient for the reduced form of 20 mM^−1^ cm^−1^ at 552 nm (Sommer et al. 2002). The actual absorbances of cytochrome *c_6_* at 552 nm were calculated by subtracting the absorption value of the oxidized samples from the reduced one (Howe and Merchant 1992) by correcting the signal to that one at 530 nm.

### Gel electrophoresis and blotting

Soluble extracts and chloroplastic fractions were loaded on 10, 12 or 15% Laemmli-SDS-PAGE gel or Tricine-SDS-PAGE (Schagger 2006) and electroblotted according to standard protocols onto PVDF membranes (Cytiva Amersham Hybond, Freiburg, Germany). A Chemiluminescence Western blotting kit (Roche, Mannheim, Germany) was used for detection. Commercially available primary antibodies for CYC6 (α-CYC6, 1/2000, Agrisera AS06 202) and FNR protein (α-FNR, 1/5000, Agrisera AS15 2909) or polyclonal antibodies raised in rabbits against recombinant APX2 (α-APX2, 1/10,000, ProteoGenix, Schiltigheim, France) and plastocyanin (α-PC, 1/500, ProteoGenix) were used to develop the blots. Fluorescence detection was carried out using an iBright FL1000 Imaging System (Invitrogen by Thermo Fisher Scientific, Brussels, Belgium).

### Proteomic analyses

Cells were grown in phototrophy under low light conditions until the mid-exponential phase. A total of 10^8^ cells were harvested by centrifugation for 3 min at 1000 *g*, at room temperature. The supernatant was discarded, and the pellet was suspended in 1 ml of RadioImmunoPrecipitation Assay buffer-like buffer: triethylammonium bicarbonate (TEAB) 100 mM, NaCl 150 mM, octylphenoxypolyethoxyethanol 1%, deoxycholic acid sodium salt 0.5% SDS 0.1%, *n*-dodecyl-β-d-maltoside 0.2% and phenylmethylsulfonyl fluoride 0.5 mM EDTA, vortexed for 10 min at room temperature and centrifuged for 10 min at 4°C at 21,000 *g*. The protein concentration of the supernatant was determined using a BCA assay (Pierce™ BCA Protein Assay Kit). Reduction of 200 μl of protein solution at 1.5 μg μl^−1^ was performed with 10 mM of 1,4-dithiothreitol at 45°C for 30 min and by alkylation with 50 mM of chloroacetamide for 30 min in the dark at room temperature. Detergents were removed using the Pierce™ Detergent Removal Spin Column (0.5 ml), and the protein content was again determined using the BCA assay (Pierce™ BCA Protein Assay Kit). A total of 60 μg of proteins were precipitated overnight at 4°C with trichloroacetic acid 20%. The pellet was washed with cold acetone and dried. The pellet was resuspended in 50 mM TEAB and digested overnight at 37°C with trypsin (Pierce™ Trypsin Protease, MS Grade). The digested samples were directly used for TMT-labeling preparation. TMT labeling was performed by following the TMTsixplex Label Reagent Set, and the fractionation in six fractions was performed with the Pierce High pH Reversed-Phase Peptide Fractionation Kit. Resulting MS/MS data were processed using Sequest HT search engine within Proteome Discoverer 2.5 against a *C. reinhardtii* reference target-decoy database obtained from Uniprot (18 828 forward entries). Trypsin was specified as the cleavage enzyme, allowing up to two missed cleavages, four modifications per peptide and up to three charges. The mass error was set to 10 ppm for precursor ions and 0.1 Da for fragment ions, and considered dynamic modifications were +15.99 Da for oxidized methionine and +42.011 Da for acetylation of the protein N-term. Fixed modifications were TMT (+229.162 Da) for lysine and peptide N-termini and +57.00 Da for carbamidomethyl cysteine. The FDR was calculated using Percolator, and thresholds for protein, peptide and modification sites were specified at 1%. Relative quantification was performed with the MS2 signal from TMT reporters’ ions. Quan value corrections for isotopic impurities were applied, and the co-isolation threshold was set at 50 for MS2 data. The mass spectrometry proteomics data have been deposited to the ProteomeXchange Consortium via the PRIDE (Perez-Riverol et al. 2022) partner repository with the dataset identifier PXD046605 and 10.6019/PXD046605.

## Supplementary Material

pcae019_Supp

## Data Availability

The transcriptomics data underlying this article are available in the National Center for Biotechnology Information, at https://www.ncbi.nlm.nih.gov/bioproject/, and can be accessed with BioProject ID: PRJNA994826. The mass spectrometry proteomics data underlying this article have been deposited to the ProteomeXchange Consortium via the PRIDE partner repository at http://www.ebi.ac.uk/pride with the Project accession identifier PXD046605 and the Project DOI 10.6019/PXD046605.

## References

[R1] Abdel-Ghany S.E. (2009) Contribution of plastocyanin isoforms to photosynthesis and copper homeostasis in Arabidopsis thaliana grown at different copper regimes. *Planta* 229: 767–779. 19084994 10.1007/s00425-008-0869-z

[R2] Asada K. (1999) THE WATER-WATER CYCLE IN CHLOROPLASTS: scavenging of active oxygens and dissipation of excess photons. *Ann. Rev. Plant Physiol. Plant Mol. Biol*. 50: 601–639. 15012221 10.1146/annurev.arplant.50.1.601

[R3] Bailleul B., Cardol P., Breyton C. and Finazzi G. (2010) Electrochromism: a useful probe to study algal photosynthesis. *Photosynth Res*. 106: 179–189. 20632109 10.1007/s11120-010-9579-z

[R4] Bolger A.M., Lohse M. and Usadel B. (2014) Trimmomatic: a flexible trimmer for Illumina sequence data. *Bioinformatics* 30: 2114–2120. 24695404 10.1093/bioinformatics/btu170 PMC4103590

[R5] Briggs L.M., Pecoraro V.L. and McIntosh L. (1990) Copper-induced expression, cloning, and regulatory studies of the plastocyanin gene from the cyanobacterium Synechocystis sp. PCC 6803. *Plant Mol. Biol*. 15: 633–642. 2129338 10.1007/BF00017837

[R6] Caccamo A., Vega de Luna F., Wahni K., Volkov A.N., Przybyla-Toscano J., Amelii A., et al. (2023) Ascorbate peroxidase 2 (APX2) of Chlamydomonas binds copper and modulates the copper insertion into plastocyanin. *Antioxidants* 12: 1946. 10.3390/antiox12111946 PMC10669542 38001799

[R7] Caverzan A., Passaia G., Rosa S.B., Ribeiro C.W., Lazzarotto F. and Margis-Pinheiro M. (2012) Plant responses to stresses: role of ascorbate peroxidase in the antioxidant protection. *Genet. Mol. Biol*. 35: 1011–1019. 23412747 10.1590/s1415-47572012000600016 PMC3571416

[R8] Chen C., Galon Y., Rahmati Ishka M., Malihi S., Shimanovsky V., Twito S., et al. (2021) ASCORBATE PEROXIDASE6 delays the onset of age-dependent leaf senescence. *Plant Physiol*. 185: 441–456. 33580795 10.1093/plphys/kiaa031 PMC8133542

[R9] Chen C., Letnik I., Hacham Y., Dobrev P., Ben-Daniel B.H., Vankova R., et al. (2014) ASCORBATE PEROXIDASE6 protects Arabidopsis desiccating and germinating seeds from stress and mediates cross talk between reactive oxygen species, abscisic acid, and auxin. *Plant Physiol*. 166: 370–383. 25049361 10.1104/pp.114.245324 PMC4149721

[R10] Cline K. (2015) Mechanistic aspects of folded protein transport by the twin arginine translocase (Tat). *J. Biol. Chem*. 290: 16530–16538. 25975269 10.1074/jbc.R114.626820 PMC4505407

[R11] Drepper F., Hippler M., Nitschke W. and Haehnel W. (1996) Binding dynamics and electron transfer between plastocyanin and photosystem I. *Biochemistry* 35: 1282–1295. 8573585 10.1021/bi951471e

[R12] Dunand C., Mathe C., Lazzarotto F., Margis R. and Margis-Pinheiro M. (2011) Ascorbate peroxidase-related (APx-R) is not a duplicable gene. *Plant Signal Behav* 6: 1908–1913. 22231200 10.4161/psb.6.12.18098 PMC3337176

[R13] Emonds-Alt B., Coosemans N., Gerards T., Remacle C. and Cardol P. (2017) Isolation and characterization of mutants corresponding to the MENA, MENB, MENC and MENE enzymatic steps of 5ʹ-monohydroxyphylloquinone biosynthesis in Chlamydomonas reinhardtii. *Plant J*. 89: 141–154. 27612091 10.1111/tpj.13352 PMC5299476

[R14] Ernst O. and Zor T. (2010) Linearization of the bradford protein assay. *J. Vis. Exp*. 38: 1918. 10.3791/1918 PMC3164080 20386536

[R15] Foyer C.H. and Noctor G. (2011) Ascorbate and glutathione: the heart of the redox hub. *Plant Physiol*. 155: 2–18. 21205630 10.1104/pp.110.167569 PMC3075780

[R16] Giraud E., Ho L.H., Clifton R., Carroll A., Estavillo G., Tan Y.F., et al. (2008) The absence of ALTERNATIVE OXIDASE1a in Arabidopsis results in acute sensitivity to combined light and drought stress. *Plant Physiol*. 147: 595–610. 18424626 10.1104/pp.107.115121 PMC2409015

[R17] Goodstein D.M., Shu S., Howson R., Neupane R., Hayes R.D., Fazo J., et al. (2012) Phytozome: a comparative platform for green plant genomics. *Nucleic Acids Res*. 40: D1178–1186. 22110026 10.1093/nar/gkr944 PMC3245001

[R18] Grabherr M.G., Haas B.J., Yassour M., Levin J.Z., Thompson D.A., Amit I., et al. (2011) Full-length transcriptome assembly from RNA-Seq data without a reference genome. *Nat. Biotechnol*. 29: 644–652. 21572440 10.1038/nbt.1883 PMC3571712

[R19] Harris E.H. (2009) Chapter 8-Chlamydomonas in the laboratory. *In* *The Chlamydomonas Sourcebook*, 1, 2nd edn. Edited by Harris E.H., Stern D.B. and Witman G.B.. pp. 241–302. Academic Press, USA.

[R20] Heinnickel M.L., Alric J., Wittkopp T., Yang W., Catalanotti C., Dent R., et al. (2013) Novel thylakoid membrane GreenCut protein CPLD38 impacts accumulation of the cytochrome b6f complex and associated regulatory processes. *J. Biol. Chem*. 288: 7024–7036. 23303190 10.1074/jbc.M112.427476 PMC3591612

[R21] Hill K.L. and Merchant S. (1992) In vivo competition between plastocyanin and a copper-dependent regulator of the Chlamydomonas reinhardtii cytochrome c(6) gene. *Plant Physiol*. 100: 319–326. 16652963 10.1104/pp.100.1.319 PMC1075554

[R22] Howe G. and Merchant S. (1992) The biosynthesis of membrane and soluble plastidic c-type cytochromes of Chlamydomonas reinhardtii is dependent on multiple common gene products. *Embo J*. 11: 2789–2801. 1322289 10.1002/j.1460-2075.1992.tb05346.x PMC556758

[R23] Hutner, S.H., Provasoli, L., Schatz, A. and Haskins, C.P. (1950) Some approaches to the study of the role of metals in the metabolism of microorganisms. *Proc. Am. Phil. Soc.* 94: 152–170.

[R24] Jia, F., Kacira, M. and Ogden, K.L. (2015) Multi-wavelength based optical density sensor for autonomous monitoring of microalgae. *Sensors.* 15: 22234–22248. 26364640 10.3390/s150922234 PMC4610439

[R25] Kitajima M. and Butler W.L. (1975) Quenching of chlorophyll fluorescence and primary photochemistry in chloroplasts by dibromothymoquinone. *Biochim. Biophys. Acta* 376: 105–115. 1125215 10.1016/0005-2728(75)90209-1

[R26] Kropat J., Gallaher S.D., Urzica E.I., Nakamoto S.S., Strenkert D., Tottey S., et al. (2015) Copper economy in Chlamydomonas: prioritized allocation and reallocation of copper to respiration vs. photosynthesis. *Proc. Natl. Acad. Sci. U.S.A*. 112: 2644–2651. 25646490 10.1073/pnas.1422492112 PMC4352834

[R27] Langmead B. and Salzberg S.L. (2012) Fast gapped-read alignment with Bowtie 2. *Nat. Methods* 9: 357–359. 22388286 10.1038/nmeth.1923 PMC3322381

[R28] Lazzarotto F., Menguer P.K., Del-Bem L.E., Zamocky M. and Margis-Pinheiro M. (2021a) Ascorbate peroxidase neofunctionalization at the origin of APX-R and APX-L: evidence from basal archaeplastida. *Antioxidants (Basel)*. 10. 10.3390/antiox10040597 PMC8069737 33924520

[R29] Lazzarotto F., Teixeira F.K., Rosa S.B., Dunand C., Fernandes C.L., de Vasconcelos Fontenele A., et al. (2011) Ascorbate peroxidase-related (APx-R) is a new heme-containing protein functionally associated with ascorbate peroxidase but evolutionarily divergent. *New Phytol*. 191: 234–250. 21352234 10.1111/j.1469-8137.2011.03659.x

[R30] Lazzarotto F., Turchetto-Zolet A.C. and Margis-Pinheiro M. (2015) Revisiting the non-animal peroxidase superfamily. *Trends Plant Sci*. 20: 807–813. 26463217 10.1016/j.tplants.2015.08.005

[R31] Lazzarotto F., Wahni K., Piovesana M., Maraschin F., Messens J. and Margis-Pinheiro M. (2021b) Arabidopsis APx-R is a plastidial ascorbate-independent peroxidase regulated by photomorphogenesis. *Antioxidants (Basel)*. 10: 1–15. 10.3390/antiox10010065 PMC7825652 33430242

[R32] Li B. and Dewey C.N. (2011) RSEM: accurate transcript quantification from RNA-Seq data with or without a reference genome. *BMC Bioinf*. 12: 1–16. 10.1186/1471-2105-12-323 PMC3163565 21816040

[R33] Li H.H. and Merchant S. (1992) Two metal-dependent steps in the biosynthesis of Scenedesmus obliques plastocyanin. Differential mRNA accumulation and holoprotein formation. *J. Biol. Chem*. 267: 9368–9375. 1577764

[R34] Li H.H. and Merchant S. (1995) Degradation of plastocyanin in copper-deficient Chlamydomonas reinhardtii. Evidence for a protease-susceptible conformation of the apoprotein and regulated proteolysis. *J. Biol. Chem*. 270: 23504–23510. 7559514 10.1074/jbc.270.40.23504

[R35] Li X., Patena W., Fauser F., Jinkerson R.E., Saroussi S., Meyer M.T., et al. (2019) A genome-wide algal mutant library and functional screen identifies genes required for eukaryotic photosynthesis. *Nat. Genet*. 51: 627–635. 30886426 10.1038/s41588-019-0370-6 PMC6636631

[R36] Li X., Zhang R., Patena W., Gang S.S., Blum S.R., Ivanova N., et al. (2016) An indexed, mapped mutant library enables reverse genetics studies of biological processes in Chlamydomonas reinhardtii. *Plant Cell* 28: 367–387. 26764374 10.1105/tpc.15.00465 PMC4790863

[R37] Love M.I., Huber W. and Anders S. (2014) Moderated estimation of fold change and dispersion for RNA-seq data with DESeq2. *Genome Biol*. 15: 1–21. 10.1186/s13059-014-0550-8 PMC4302049 25516281

[R38] Mason C.B., Bricker T.M. and Moroney J.V. (2006) A rapid method for chloroplast isolation from the green alga Chlamydomonas reinhardtii. *Nat. Protoc*. 1: 2227–2230. 17406461 10.1038/nprot.2006.348

[R39] Maxwell K. and Johnson G.N. (2000) Chlorophyll fluorescence—a practical guide. *J. Exp. Bot*. 51: 659–668. 10938857 10.1093/jxb/51.345.659

[R40] Merchant S. and Bogorad L. (1987) The Cu(II)-repressible plastidic cytochrome c. Cloning and sequence of a complementary DNA for the pre-apoprotein. *J. Biol. Chem*. 262: 9062–9067. 3036842

[R41] Newman S.M., Boynton J.E., Gillham N.W., Randolph-Anderson B.L., Johnson A.M. and Harris E.H. (1990) Transformation of chloroplast ribosomal RNA genes in Chlamydomonas: molecular and genetic characterization of integration events. *Genetics* 126: 875–888. 1981764 10.1093/genetics/126.4.875 PMC1204285

[R42] Perez-Riverol Y., Bai J., Bandla C., Garcia-Seisdedos D., Hewapathirana S., Kamatchinathan S., et al. (2022) The PRIDE database resources in 2022: a hub for mass spectrometry-based proteomics evidences. *Nucleic Acids Res*. 50: D543–D552. 34723319 10.1093/nar/gkab1038 PMC8728295

[R43] Pesaresi P., Scharfenberg M., Weigel M., Granlund I., Schroder W.P., Finazzi G., et al. (2009) Mutants, overexpressors, and interactors of Arabidopsis plastocyanin isoforms: revised roles of plastocyanin in photosynthetic electron flow and thylakoid redox state. *Mol. Plant* 2: 236–248. 19825610 10.1093/mp/ssn041

[R44] Pierre Y., Breyton C., Kramer D. and Popot J.L. (1995) Purification and characterization of the cytochrome b6 f complex from Chlamydomonas reinhardtii. *J. Biol. Chem*. 270: 29342–29349. 7493968 10.1074/jbc.270.49.29342

[R45] Ritchie R.J. (2006) Consistent sets of spectrophotometric chlorophyll equations for acetone, methanol and ethanol solvents. *Photosynth Res*. 89: 27–41. 16763878 10.1007/s11120-006-9065-9

[R46] Schagger H. (2006) Tricine-SDS-PAGE. *Nat. Protoc*. 1: 16–22. 17406207 10.1038/nprot.2006.4

[R47] Shigeoka S., Ishikawa T., Tamoi M., Miyagawa Y., Takeda T., Yabuta Y., et al. (2002) Regulation and function of ascorbate peroxidase isoenzymes. *J. Exp. Bot*. 53: 1305–1319. 11997377

[R48] Sommer F., Drepper F. and Hippler M. (2002) The luminal helix l of PsaB is essential for recognition of plastocyanin or cytochrome c6 and fast electron transfer to photosystem I in Chlamydomonas reinhardtii. *J. Biol. Chem*. 277: 6573–6581. 11744732 10.1074/jbc.M110633200

[R49] Strasser R.J. Govindjee (1992) The Fo and the O-J-I-P fluorescence rise in higher plants and algae. *In* *Regulation of Chloroplast Biogenesis*, *NATO Science Series AI series*. 226, 1. Edited by Argyroudi-Akoyunoglou J.H. pp. 423–426. Springer, Boston, MA.

[R50] Terzulli A. and Kosman D.J. (2010) Analysis of the high-affinity iron uptake system at the Chlamydomonas reinhardtii plasma membrane. *Eukaryot. Cell*. 9: 815–826. 20348389 10.1128/EC.00310-09 PMC2863958

[R51] Tyagi S., Verma P.C., Singh K., Upadhyay S.K. and Upadhyay S.K. (2020) Molecular characterization of ascorbate peroxidase (APX) and APX-related (APX-R) genes in Triticum aestivum L. *Genomics* 112: 4208–4223. 32681868 10.1016/j.ygeno.2020.07.023

[R52] Verma D., Upadhyay S.K. and Singh K. (2022) Characterization of APX and APX-R gene family in Brassica juncea and B. rapa for tolerance against abiotic stresses. *Plant Cell Rep*. 41: 571–592. 34115169 10.1007/s00299-021-02726-0

[R53] Weigel M., Varotto C., Pesaresi P., Finazzi G., Rappaport F., Salamini F., et al. (2003) Plastocyanin is indispensable for photosynthetic electron flow in Arabidopsis thaliana. *J. Biol. Chem*. 278: 31286–31289. 12773541 10.1074/jbc.M302876200

[R54] Zecha J., Satpathy S., Kanashova T., Avanessian S.C., Kane M.H., Clauser K.R., et al. (2019) TMT labeling for the masses: a robust and cost-efficient, in-solution labeling approach. *Mol. Cell. Proteomics* 18: 1468–1478. 30967486 10.1074/mcp.TIR119.001385 PMC6601210

[R55] Zivcak M., Brestic M., Kunderlikova K., Olsovska K. and Allakhverdiev S.I. (2015) Effect of photosystem I inactivation on chlorophyll a fluorescence induction in wheat leaves: does activity of photosystem I play any role in OJIP rise? *J. Photochem. Photobiol. B* 152: 318–324. 26388470 10.1016/j.jphotobiol.2015.08.024

