# Supplementary material for: APX2 Is an Ascorbate Peroxidase–Related Protein that Regulates the Levels of Plastocyanin in Chlamydomonas
Source: Plant Cell Physiol. 2024 Mar 2;65(4):644–56. doi: 10.1093/pcp/pcae019 (PMC11094752; doi:10.1093/pcp/pcae019)
Supplement: pcae019_Supp [file pcae019_supp.zip › suppl_data/pcp-2023-e-00263-File009.docx]

**Supplemental figures**

**Figure S1. Uncropped gel images of the immunoblots showed in Fig. 1, 3, 4 and 5**

**A**) Uncropped immunoblot, as shown in **Fig. 1B,** displays a serial dilution of soluble protein extracts fractions of wt, compared with the *apx2-1* and *apx2-2* mutants, labelled using APX2 antibodies. Coomassie blue staining of the gel serves as a loading control. The 24 kDa band corresponds to APX2. The letters 'a' and 'b' indicate unspecific bands. **B**) Uncropped immunoblot, as shown in **Fig. 1C,** displays chloroplast fractions of wt and *apx2-1* labelled with APX2 and FNR antibodies. Coomassie blue staining of the gel serves as control of loading. The black line in each panel indicates the separation on the blot/gel between the ladder and the loadings of wt and *apx2-1*, signifying the removal of some samples unrelated to the study. **C**) Uncropped immunoblot, as shown in **Fig. 3E,** exhibits a serial dilution of soluble protein extracts of wt compared with the *apx2-1* and *apx2-2* mutants labelled with plastocyanin (PC) and cytochrome *f* (Cyt*f*) antibodies. Please note that the Cyt*f* immunoblot is a rehybridization after the PsaD labelling (see panel **D**). Coomassie blue staining of the gel is shown as a loading control. **D**) Uncropped immunoblot as shown in **Fig. 4B** of serial dilution of soluble protein extracts of wt compared with the *apx2-1* and *apx2-2* mutants labelled with PsaF and PsaD antibodies. Coomassie blue staining of the gel serves as a loading control. **E**) Uncropped immunoblot, as shown **Fig. 4C,** shows a serial dilution of soluble protein extracts of wt compared with the *apx2-1* and *apx2-2* mutants labelled with multicopper ferroxidase (FOX1) antibodies. Coomassie blue staining of the gel serves as a loading control. **F**) Uncropped immunoblot, as shown in **Fig. 5B,** features soluble protein extracts of wt, *apx2-1* and *apx2-2* mutants labelled with cytochrome *c_6_* (CYC6) antibodies. Coomassie blue staining of the gel is shown as a loading control.


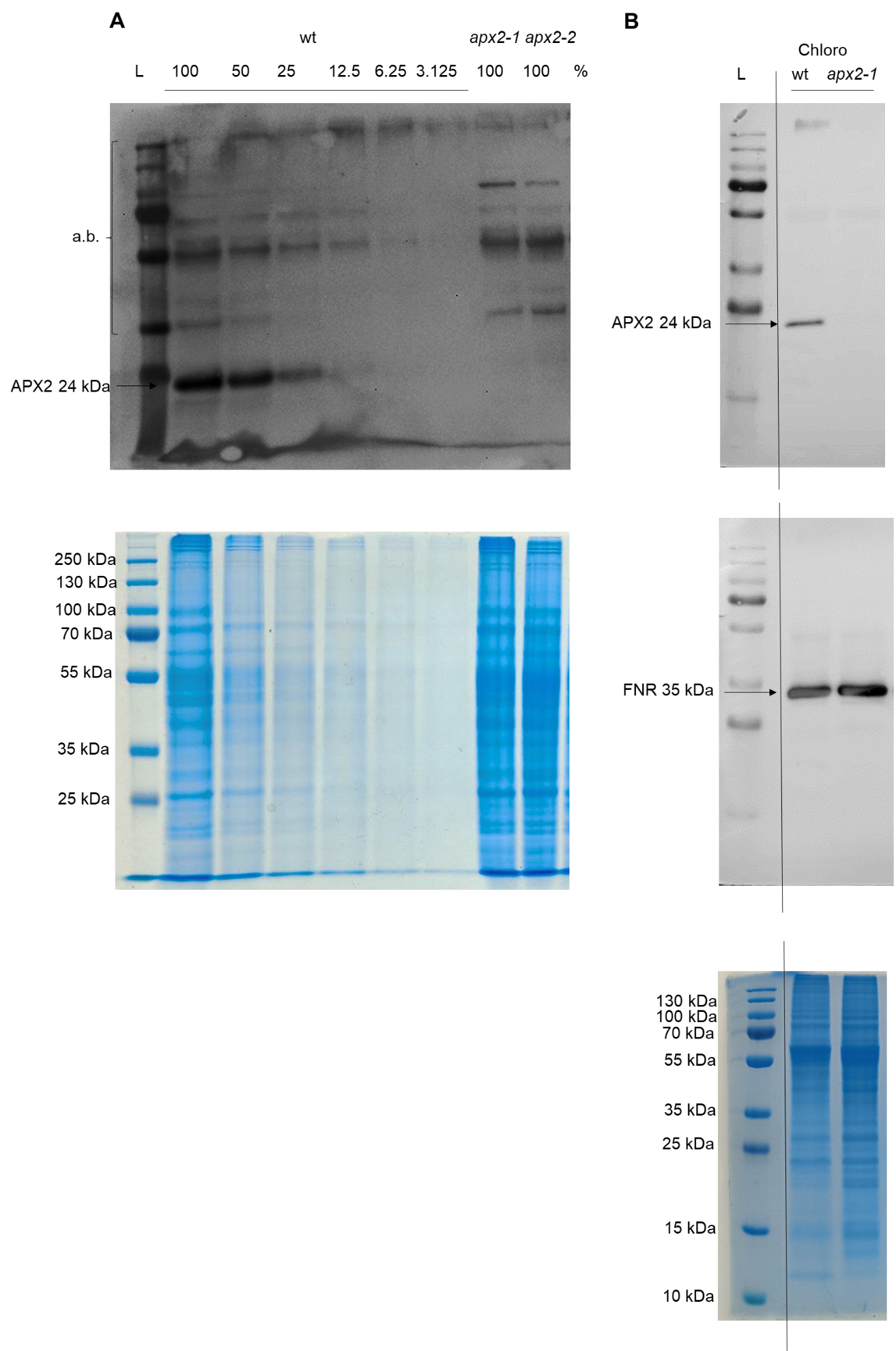


**
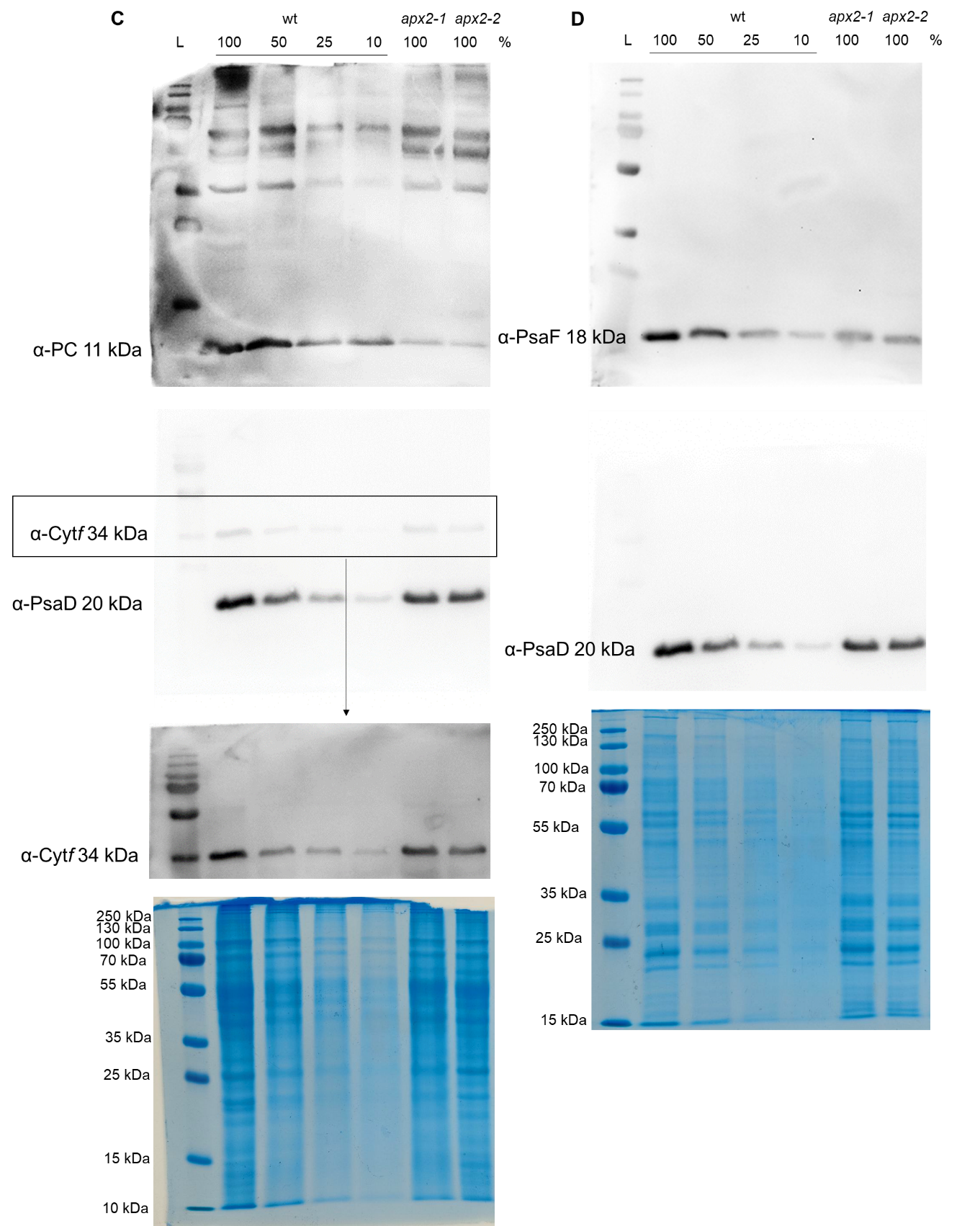
**

**
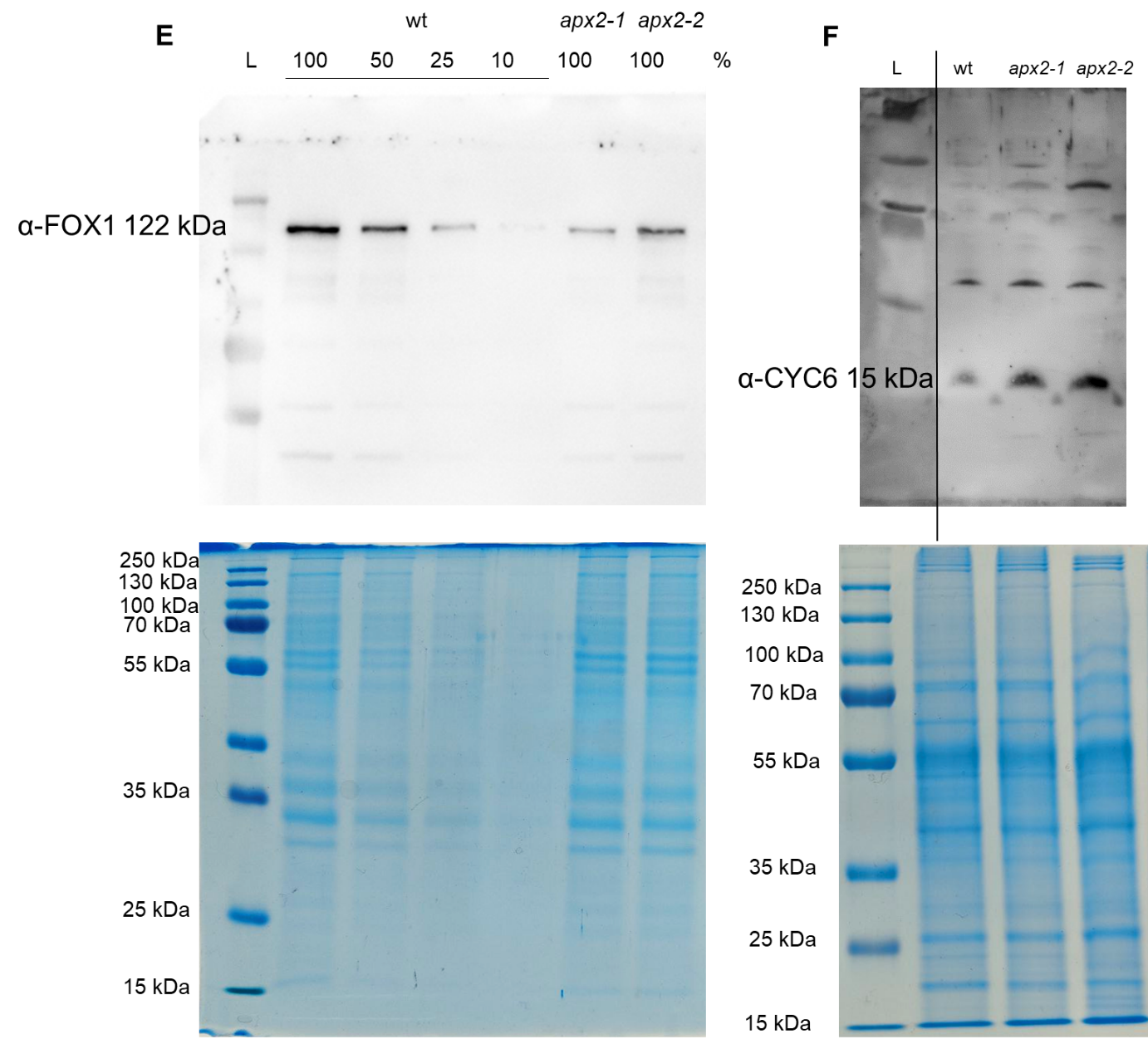
**

**Figure S2. Wt and *apx2-1* exhibit diverse responses to copper deficiency, with the *APX2* transcript remaining unaffected in the presence of copper deficiency**

**A**) The heat map illustrates the 50 most up-regulated genes and the 20 most down-regulated genes in each comparison for both wild type (wt) and *apx2-1* mutants, under copper-replete (6 μM CuSO_4_) and copper-deficient conditions (1 nM CuSO_4_). Variations are observed within the strains. Genes with a log_2_ fold change < -1 (range violet – black) represent down-regulated ones, while those with a log_2_ fold change > 1 (range pink – cream) signify up-regulated ones, all with a p-value < 0.05, as analysed by DESeq2. Rows are labelled with gene symbols (if available) or gene locus. The three columns represent the following comparisons: wt vs. *apx2-1* in control conditions with standard copper concentration (wt-apx2), wt in control vs. copper-deficient conditions (wt-wt [1 nM Cu]), and *apx2-1* in control vs. copper-deficient conditions (apx2-apx2 [1 nM Cu]). **B**) Normalized reads for the *APX2* transcript demonstrate that gene expression is independent of copper. Three replicates of wt in copper-replete and in copper-deficient conditions are presented.


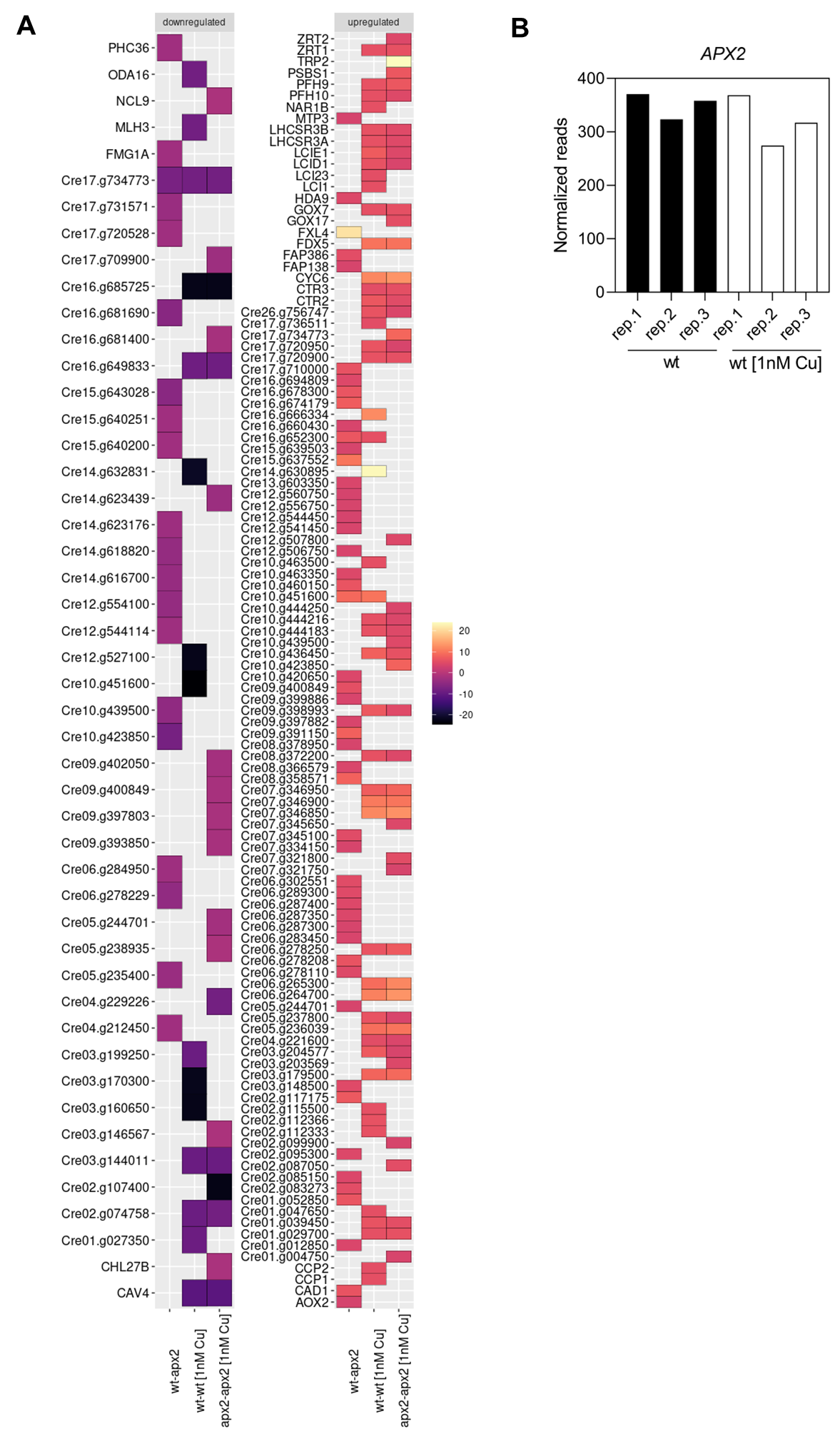


**Figure S3. Alignment of Arabidopsis APX6 and Chlamydomonas APX2 reveals the absence of the AxA cleavage site of the TAT signal in APX6 but confirms the presence of the MxxM motif.**The double arginine (RR) of the TAT signal is highlighted in pink, and the hydrophobic region in grey. The cleavage site is only found in Chlamydomonas (putative AAA, highlighted in bold). The MxxM motif is in bold orange.

**Supplemental tables**

**Table S1.** **Confirmation of the presence of paromomycin cassette in the three *apx2* mutants**

| **Mutant name** | **CLiP reference^1^** | **PCR** | **Sequencing with** | **Sequence^2^** |
| --- | --- | --- | --- | --- |
| *apx2-1* | LMJ.RY0402.180063 | *apx2-1*_R  OMJ913 | *apx2-1*_R | GCACCAATCATGTCAAGCCTCAGCGAGCTGCCTGATGGATGGTTCTCTAGCTTGCCCCTGACCGGACGCGTGCTGGTAGGAGACGTGTTTCTGACGAGGGCTCGTGACGAAGGTGCGTGAGAGGGTGCGAGTTACGAGGACCAAATTTGCAAGGGTGTGCAACAGTGGCGGGTCTACTTGGGGCAGTGGGGGAAGCAGGGCGCAGCGGGTGGGCGAGGCAGGACGGTGCCACTTTGCGGAGGCACGGCACGGCGCCTCAGGGCCGCGCTGGTGAGTGGTGCCCCCACCATCCCGGTCCGGTGTTGGTGATCTGGATGCGGCTCAGTTCGCATGATGCGCCCGGATGCCCTCTGTGCGCACCATTATGACC |
|  |  | *apx2-1*_F  OMJ944 | *apx2-1*_F | TTTGACGTTACCAGCACACCCTTGATCATCATCAGCTGCTCTTCCCTGCCGCTGCAACACGCCCCGCGCTACGCCGGTCTGTCGGAACTGCACACTGACGTCGAGCCTTCTGGCAGACTAGTTGCTCCTGAGTCCAACGATGTTCTTAGCAAGAGCCTTGTCCAACGCAGCCGCCACGGCCGGAGACGGCAACGTCGGCCCCTCGGCGGCGGCCGGCAGTGGCGCCAGCAGCAGCGCCGGCGGGGTGGCCGCGGCCAGAGCCGCCGCCAGGGCCGGCAGTGCCGCCCCCAGCTTCAGCACCGCTCGTCGCGAAAGCTCCGGAGCTACAGCCGGCTCCGAGGTACCGCTGCCTGTCCTAGTCAGGCGTGACGCAACCGCGATGCCCGGGGCGCGACCGTGACGGCAGGCAACGACGTACGTACTAGTTACTAGCTATCTAAGAGCTCGCATCCCCTGCGCTCGCCTCCCTCCGGTGCATGCGCCAGTTGCCGGCGGGGCATCGTGGCCACGGGTACCATCCCGAGGGGTACAGTTACGCCCGAGCTGCGGCCCATCGAACATCCGCCACGCACCCTCTTGGCGCCACTGTGACAGGTCCCGTAGCTTGGCATGCGTCGCAACTCTGCACGCTTGCGCTTGCCGG |
| *apx2-2* | LMJ.RY0402.095128 | *apx2-2*_F  OMJ944 | OMJ944 | TATGCAGCTCTCTGCCGCTGCACACGCCCCGCGCTATAGCCGCACCGCTAGTATGGTAACTGACGTCGAGCCTTCTGGCAGACTAGTTGCTCCTGAGTCCAACTCTGCACGCTCGTCGCGGGTTGCCATGGCGTGTGCAATTCGACAGGGAACGGTGCGAAAGGACGCTTGTACTATGTTGTGACTACGGTGCGAAAGGACGCTTGTACTAAAGGGAAAGTCCCATGGTTAACCTGGTTTTGGCCCTGATTTTGCCGCATACACGGGATCGGCGTGAGCCTCAAGGGCGTGGGTGTTGCTTGCTTCGCCCACTCTTCAGTCCGCGGTTACGCTTAGCGAGCCTACACGGTCGGCTAGGGCGAAAGGAAGCTTGCAAAAAACGCATGTCACCCAGCTGCGATCTTGTGTATGGCCAGGATTTTTTTTTTAGTAATGT |

^1^<https://www.chlamylibrary.org/>

^2^ yellow: internal bar code of the cassette (<https://www.chlamylibrary.org/>); pink: paromomycin resistant cassette (CIB1, https://www.chlamylibrary.org/); grey: APX2 sequence.

**Table S2. Primers used for sequencing**

| **Mutants** | **Primer name** | **Orientation 5’->3’** |
| --- | --- | --- |
| *apx2-1* | *apx2-1*_F | TTCCCTGTCGAATTGCAC |
|  | *apx2-1*_R | CGAGACCACACATATCAAGG |
| *apx2-2* | *apx2-2*_F | GAGTACAAGCGTCCTTTCGCAC |
|  | *apx2-2*_R | GGGGATGTTCTTAGCAAGAGCC |
|  | | |
| cassette | OMJ913 | GCACCAATCATGTCAAGCCT |
| cassette | OMJ944 | GACGTTACAGCACACCCTTG |
